# Relationship of smoking status to genomic profile, chemotherapy response and clinical outcome in patients with advanced urothelial carcinoma

**DOI:** 10.18632/oncotarget.9449

**Published:** 2016-05-18

**Authors:** Monika Joshi, Monali Vasekar, Petros Grivas, Hamid Emamekhoo, JoAnn Hsu, Vincent A. Miller, Philip J. Stephens, Siraj M. Ali, Jeffrey S. Ross, Junjia Zhu, Joshua Warrick, Joseph J. Drabick, Sheldon L. Holder, Matthew Kaag, Min Li, Sumanta Kumar Pal

**Affiliations:** ^1^ Department of Medicine, Division of Hematology/Medical Oncology, Penn State Hershey Cancer Institute, Hershey, PA, USA; ^2^ Department of Hematology/Medical Oncology, Cleveland Clinic Taussig Cancer Institute, Cleveland, OH, USA; ^3^ Department of Medical Oncology, City of Hope Comprehensive Cancer Center, Duarte, CA, USA; ^4^ Foundation Medicine, Cambridge, MA, USA; ^5^ Department of Pathology, Albany Medical College, Albany, NY, USA; ^6^ Department of Pathology, Penn State Hershey Cancer Institute, Hershey, PA, USA; ^7^ Department of Surgery, Division of Urology, Penn State Hershey Cancer Institute, Hershey, PA, USA; ^8^ Department of Biostatistics, City of Hope Comprehensive Cancer Center, Duarte, CA, USA

**Keywords:** advanced, chemotherapy, metastatic, genomic profiling, smoking

## Abstract

Smoking has been linked to urothelial carcinoma (UC), but the implications on genomic profile and therapeutic response are poorly understood. To determine how smoking history impacts genomic profile and chemotherapy response, clinicopathologic data was collected for patients with metastatic UC (mUC) across 3 academic medical centers and comprehensive genomic profiling (CGP) was performed through a CLIA-certified lab. Unsupervised hierarchical clustering based on smoking status was used to categorize the frequency of genomic alterations (GAs) amongst current smokers (CS), ex-smokers (ES) and non-smokers (NS), and survival was compared in these subsets. Fisher's exact test identified significant associations between GAs and smoking status. Amongst 83 patients, 23%, 55% and 22% were CS, ES, and NS, respectively, and 95% of patients had stage IV disease. With a median follow up of 14.4 months, the median overall survival (OS) was significantly higher in NS and ES (combined) as compared to CS (51.6 vs 15.6 months; *P* = 0.04). Of 315 cancer-related genes and 31 genes often related to rearrangement tested, heatmaps show some variations amongst the subsets. GAs in NSD1 were more frequent in CS as compared to other groups (*P* < 0.001). CS status negatively impacts OS in patients with mUC and is associated with genomic alterations that could have therapeutic implications.

## INTRODUCTION

Smoking is a well-established risk factor for urothelial carcinoma (UC), and roughly three-quarters of patients with this disease have a smoking history [1]. In several diseases, smoking history has important biological and clinical implications. For example, in advanced lung cancer, smoking status is linked to higher mutational burden, higher rates of C > A transversion mutations, and increased frequency of *EGFR* alterations, the latter having subsequent impact on selection of EGFR-directed therapies [2–4]. Thus far, smoking history has played little role in treatment allocation for UC.

Recently, several datasets have emerged to more completely characterize the genomic landscape of UC. The Cancer Genome Atlas (TCGA) investigators have published data from 131 patients with UC, with potential therapeutic targets identified in roughly 69% [5]. The most frequently encountered genomic alterations (GAs) in this series were in the phosphatidylinositol-3-OH kinase/AKT/mTOR pathway (42%) and RTK/MAPK pathway (45%). An updated publication of TCGA data from a total of 412 patients with UC is forthcoming [6]. A limitation of this work, however, is the lack of clinical outcome and follow-up data. We have previously reported the comprehensive genomic profile (CGP) of 295 patients with advanced UC assessed using a CLIAA-certified assay (Foundation Medicine; Cambridge, MA) [7]. In this cohort of patients with largely stage IV disease, even higher frequencies of clinically relevant GAs (CRGAs) were identified (93%). This series was also limited by a lack of clinical annotation, making it a challenge to convey associations between CRGAs and clinical outcome. We have previously reported CGP in 69 patients with UC of bladder where PIK3CA mutation appeared to be more frequent in non-smokers when compared to current and ex-smokers (43% vs. 11%, *p* = 0.1760). The study was limited due to small sample size with majority being non-metastatic (20% of patients had stage IV disease) and lack of uniformity of genomic sequencing for all patients [8].

In the current manuscript, we identify a cohort of mostly metastatic UC patients who had CGP performed in the context of either routine care or screening evaluation for prospective clinical trials. With detailed clinical annotation available for each patient, we evaluated the association between smoking status and clinical outcome (e.g., response to chemotherapy and overall survival [OS]). Furthermore, we associate smoking status with results of CGP.

## RESULTS

### Clinicopathologic data

A total of 83 patients were assessed across the 3 academic sites. The male: female ratio in the cohort was 3:1, and the median age was 62 (range, 44–84) (Table 1). Seventy-nine patients (95%) had stage IV disease; the remaining 4 patients had advanced disease (stage III). Of patients with stage IV disease, 28 patients (35%) had *de novo* metastatic disease. A total of 47 patients received platinum-based chemotherapy (defined as a regimen containing either cisplatin or carboplatin) in the first-line setting, and 56 patients had received prior cystectomy. In total, 18 (22%), 46 (55%) and 19 (23%) patients were characterized as current smokers, ex-smokers and non-smokers, respectively.

**Table 1:** Patient characteristics

	Current Smokers	Ex-Smokers	Non-Smokers
	(*n* = 18)	(*n* = 46)	(*n* = 19)
Gender, *n* (%)			
Female	1 (5.6%)	8 (17.4%)	9 (47.4%)
Male	17 (94.4%)	38 (82.6%)	10 (52.6%)
Age, median (IQR) †	62 (48–74)	67 (44–84)	60 (45–83)
Race			
Caucasian	15 (83.3%)	39 (84.8%)	17 (89.5%)
Black	2 (11.1%)	3 (6.6%)	0 (0%)
Asian	0 (0%)	2 (4.3%)	2 (10.5%)
Other	1 (5.6%)	2 (4.3%)	0 (0%)
Prior Pelvic Radiation			
Yes	6 (33.3%)	10 (21.7%)	2 (10.5%)
No	12 (66.7%)	36 (78.3%)	17 (89.5%)
Prior Cystectomy			
Yes	10 (55.6%)	29 (63.0%)	17 (89.5%)
No	8 (44.4%)	17 (37.0%)	2 (10.5%)
Histology			
Pure urothelial	13 (72.2%)	25 (54.3%)	16 (84.2%)
Mixed	0 (0%)	4 (8.7%)	1 (5.3%)
Not Available	5 (27.8%)	17 (37.0%)	2 (10.5%)
Neoadjuvant chemo			
Yes	5 (27.8%)	13 (28.3%)	6 (31.6%)
No	13 (72.2%)	33 (71.7%)	13 (68.4%)
Adjuvant chemo			
Yes	2 (11.1%)	2 (4.3%)	4 (21.1%)
No	16 (88.9%)	44 (95.7%)	15 (78.9%)
Lines of therapy for metastatic disease			
0	1 (5.6%)	8 (17.4%)	2 (10.5%)
1	5 (27.8%)	13 (28.3%)	6 (31.6%)
2	6 (33.3%)	13 (28.3%)	8 (42.1%)
3	6 (33.3%)	5 (10.9%)	2 (10.5%)
> 3	0 (0%)	7 (15.1%)	1 (5.3%)

### Genomic differences by smoking status

**Figure 1:**
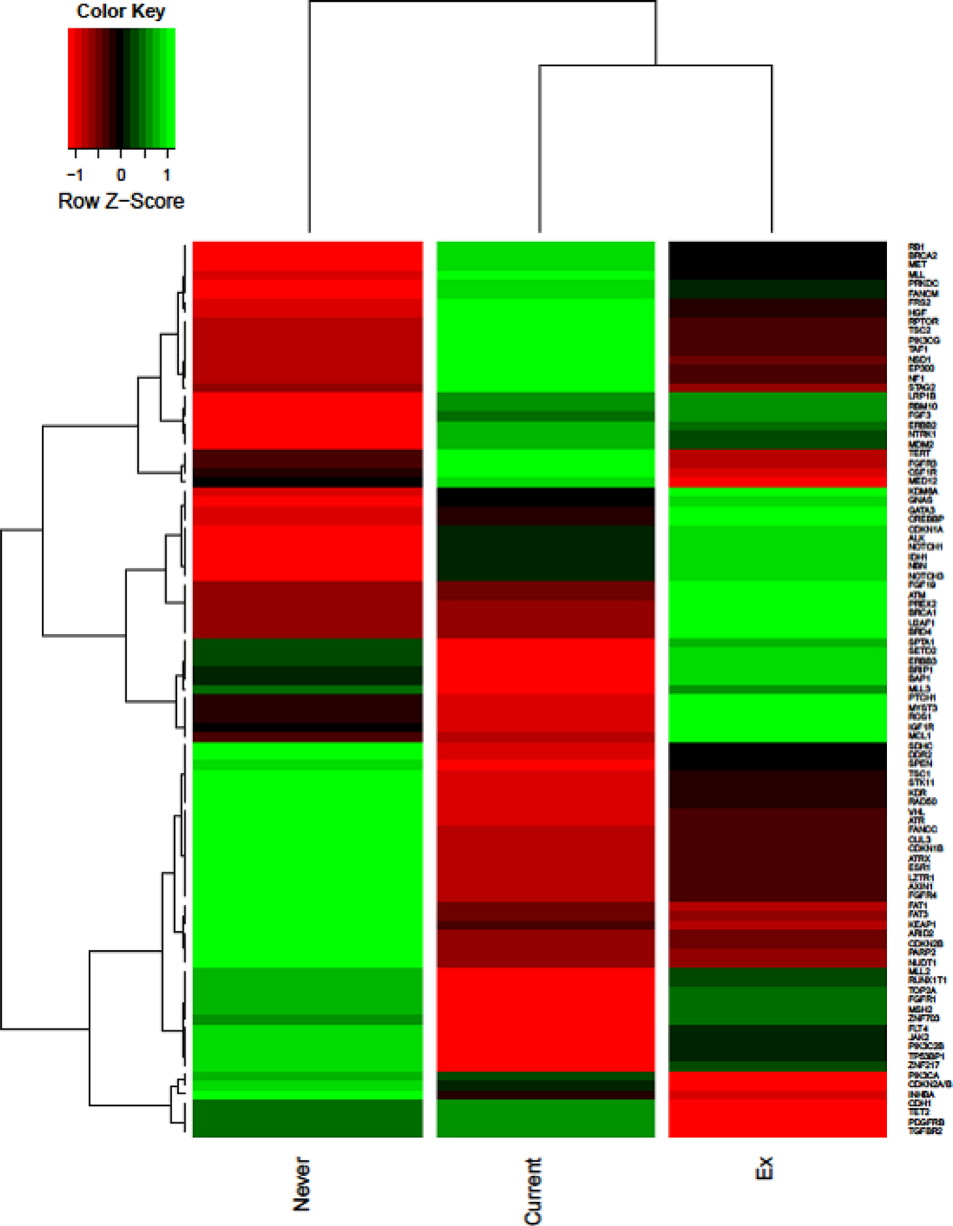
Heatmap delineating GAs in advanced bladder cancer patients with unsupervised hierarchical clustering based on smoking status, shown for 93 genes with a minimum of 7.5% difference in GA frequencies amongst smoking status

CGP data was available for all 83 patients in the cohort. Of these tissues, 36 (43.3%) were derived from cystectomy/nephroureterectomy specimens, 26 (31.3%) were derived from TURBT (transurethral resection of bladder) specimens, 4 (4.8%) were derived from lymph node site at the time of surgery, and 16 (19.2%) were derived from metastatic site. The origin of tissue for 1 patient was missing. Majority of the patients (89%) had their tissue analyzed from the archival tissue prior to treatment with chemotherapy. A heat-map with unsupervised hierarchical clustering based on smoking status is shown in Figure 1. The distance matrix was computed based on pairwise Pearson correlation. As visible in this heat-map, several genes appear to be more frequently altered in non-smokers as compared to current smokers, including targetable entities involved in DNA repair pathways (*ATR*), cell cycle (*CDKN1B* and *CDKN2B*) and mTOR signaling (*TSC1*). In contrast, current smokers had more frequent alternations in distinct DNA repair genes (*BRCA2*), epigenetic moieties (*EP300*) and other targetable signal transduction mediators (*FGFR3)*. Interestingly, current smokers and ex-smokers exhibited a distinct profile, with the latter demonstrating more frequent alterations in selected epigenetic and DNA repair moieties (*CREBBP* and *BRCA1*, respectively).

Two statistically significant differences in mutational frequency were identified. Current smokers were noted to have a higher frequency of GAs in *NSD1* as compared to ex-smokers and non-smokers (39% *vs* 7% *vs* 0%, respectively; *P* < 0.001). *TGFBR2* alterations were also more frequent in non-smokers and current smokers (11% in both groups) as compared to ex-smokers (0%; *P* = 0.04). Differences in mutational frequency among smoking groups were identified in several other genes, though none reached statistical significance.

Notably, no significant changes were noted in the cumulative number of mutations detected (a surrogate for mutational load) amongst subgroups divided by smoking status, with an average of 19.5, 20.5, and 17.3 mutations in current smokers, ex-smokers and non-smokers, respectively.

### Differences in clinical outcome by smoking status

**Figure 2:**
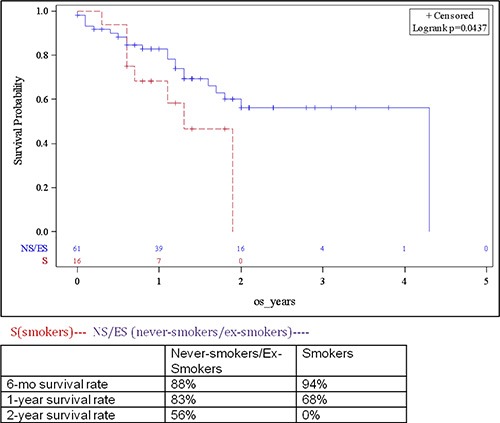
Survival of patients with advanced bladder cancer based on smoking status (landmark analyses presented in table insert)

Of patients with stage IV disease, 47 had received platinum-based chemotherapy in the front-line setting. Amongst evaluable patients, the overall response rate (ORR), combining complete responses (CRs) and partial responses (PRs), was 37.5% (6/16), 47% (16/34) and 19% (3/16) in current smokers, ex-smokers and non-smokers, respectively (*p* = 0.149, NS). Despite the higher response rate amongst current smokers as compared to non-smokers, the OS of patients in this cohort was lower as compared to a combined cohort comprised of ex- and non-smokers (15.6 *vs* 51.6 months; *p* = 0.04) (Figure 2).

## DISCUSSION

Despite the well-documented association between smoking and urothelial cancer, the impact of smoking on urothelial cancer biology and clinical outcome is quite poorly understood. To our knowledge, our study is one of the first to both characterize differences in genomic landscape and chemotherapy response in patients with advanced urothelial cancer based on smoking status. Smoking status appears to be associated with some profound differences in genomic profile, and could bear relevance to patient selection for trials of targeted therapy, chemotherapy and/or immunotherapy, as discussed subsequently. Current smoking appears to have a negative prognostic impact, possibly suggesting that the genomic landscape associated with this disease may result in a more aggressive phenotype.

At present, platinum-based chemotherapy remains a standard for patients with advanced urothelial carcinoma, with survival estimates ranging from 13–15 months with cisplatin-based chemotherapy [11]. Treatment with carboplatin-based regimens, although occasionally warranted for patients with renal insufficiency or other comorbidities, is generally thought to yield an even poorer outcome [12]. Beyond these approaches, there has been much enthusiasm surrounding use of novel immunotherapeutic strategies such as programmed death-1 (PD-1) inhibition or PD-L1 inhibition. Agents that block PD-1 or and its cognate ligand have response rates ranging from 10–30% following platinum-based chemotherapy, with survival that bests most cytotoxic agents in this setting [13, 14]. Luminal cluster II molecular subtype and mutational load (but not smoking status) were shown to be recently associated with higher response rate to anti-PD-L1 agent in patients with platinum-refractory advanced UC [15]. Moreover there has been a suggestion in melanoma and lung cancer that mutational load may identify patients more likely to respond to immunotherapy [16, 17]. Using the cumulative number of mutations as a surrogate for mutational load (and possibly neo-antigen formation), we noted no significant differences across cohorts divided by smoking status.

Aside from novel immunotherapeutic approaches, there is interest in using an array of targeted therapies in molecularly selected subsets of patients. In urothelial cancer, several studies and case reports have shown benefit with everolimus when applied in patients with *TSC1* specific alterations, found more commonly altered, among non-smokers in our series [18, 19]. There is also early but compelling data for use of fibroblast growth factor receptor (FGFR) inhibitors in patients with *FGFR3*-altered urothelial cancer, an alteration found more commonly in current smokers in our study [20]. Larger studies exploring these approaches are ongoing, but accrual has been challenged by poor access to genomic sequencing data in patients with advanced urothelial cancer.

Perhaps the most compelling finding from this study is the identification of a high frequency of *NSD1* alteration in current smokers, although the results should be interpreted with caution given small number of patients. *NSD1* alterations have been associated with Sotos syndrome, a syndrome of childhood overgrowth [21]. In the context of malignancy, translocations involving *NSD1* have been identified in pediatric leukemias [22]. NSD1 may also have a complex interplay with NF-kappaB, regulating the latter through methylation of p65 [23]. At present, no agents specifically targeting NSD1 exist, but presumably, hypomethylating agents such as azacitidine could be considered as a therapeutic trial strategy. A more marginal association was found between smoking status and *TGFBR2* alteration, with higher rates in both current smokers and non-smokers as compared to ex-smokers. Several different approaches are being undertaken to target transforming growth factor-beta (TGF-beta) in solid tumors, including development of antisense oligonucleotide [24].

The idea that a molecular signature or single gene mutation in urothelial carcinomas would differ between smoking-associated and smoking-independent cancers is supported by prior observations in other tumor types. For example, in lung cancer smoking-independent cancers are in many respects biologically distinct, having more frequent EGFR mutations, lower mutational burden, and lower rates of the C > A transversion mutations than smoking-associated lung cancers. [2, 3]. Our study identifies *NSD1* as possibly having differences in mutational frequency amongst smoking groups and is hypothesis generating. A larger analysis is required to rigourously interrogate genetic alterations in *NSD1* as possibly differing amongst smoking groups.

With respect to clinical outcomes based on smoking status, our findings suggest no significant differences in response rate to platinum-based chemotherapy, with a surprisingly low response rate amongst non-smokers. However, current smokers had the shortest OS. These data are challenging to interpret, in part because of our limited sample size but also due to potential confounders – it is possible that current smokers possessed other associated comorbidities (e.g. respiratory, cardiovascular disease) that could either directly impact survival or limit the ability to deliver systemic therapy. Nonetheless, our findings were consistent with recently published meta-analytic data suggesting that smoking is associated with a more aggressive subset of urothelial carcinoma with a poorer prognosis [25].

The data from the present study suggest that CS status portends worse OS in patients with advanced urothelial cancer. Our data has certainly limitations based on sample size and a lack of correction for multiple comparisons. Despite these limitations, mutations in *NSD1* were identified more frequently in current smokers compared to other smoking groups. It is possible that these may underlie the poorer clinical outcome seen in these patients. Future studies with a larger sample size will allow such results to be interpreted with greater degree of confidence.

## MATERIALS AND METHODS

### Patient selection

Using separate institutionally-approved IRB protocols, patients with metastatic UC were identified across three academic medical centers: (1) City of Hope Comprehensive Cancer Center, (2) Penn State Hershey Cancer Institute, and (3) Cleveland Clinic Taussig Cancer Institute. Only the patients who had CGP performed using a CLIAA-certified assay (described subsequently) as part of routine clinical care or for the purpose of enrollment in a prospective clinical trial were selected. Patients eligible for the study were also required to have a documented smoking status classifiable by a standard definition. Current smokers were defined as individuals who have smoked at least 100 cigarettes in their lifetime and continue to smoke. Ex-smokers were defined as individuals who smoked ≥ 100 cigarettes in the past (> 1 month since the time of diagnosis) but no longer smoked at the time of diagnosis with UC. Non-smokers were defined as individuals who had smoked less than 100 cigarettes in their lifetime or have never smoked.

### Ascertainment of CGP

Detailed methods for the CGP assay used herein have been previously published [9]. Tissue blocks or formalin-fixed, paraffin-embedded (FFPE) slides were obtained from all patients in the cohort. Patients with unusual dominant histologies (e.g., small cell, neuroendocrine or adenocarcinoma) were not included in the analysis. DNA was extracted from tissue blocks or FFPE slides and CGP based on targeted next generation sequencing (NGS) of established cancer-related genes was performed on hybridization-captured, adaptor ligation-based libraries in a CLIAA-certified lab (Foundation Medicine, Inc.; Cambridge, MA). All cases were sequenced with deep coverage across all coding exons from 315 cancer-related genes and 31 genes often related to rearrangement. Cases were sequenced to a median depth of 650x. Base substitutions, short insertions, deletions, copy number changes, gene fusions and rearrangements were assessed in a manner akin to previous reports [10]. A comprehensive list of gene alterations included in the Foundation Medicine assay has been reported by Frampton *et al.*

### Statistical analysis

For CGP data, Bayesian algorithms were used to identify substitutions and local assembly algorithms were used to detect insertions/deletions. Copy number alterations were detected through comparison to normal control samples. A CRGA was defined as a GA that could be linked to either an approved or investigational targeted agent. Pooled CGP data across patients from the 3 sites was assessed through generation of a heat-map, with unsupervised hierarchical clustering based on smoking status. The Fisher's exact test was used to compare mutational frequencies amongst subgroups, and was also used to calculate differences in response rate to platinum-based chemotherapy in the front-line setting. With respect to clinical outcomes, the Kaplan-Meier method and log-rank test were used to compare survival amongst subsets defined by smoking status. Hazard ratio (HR) is estimated by Cox proportional hazard model. Given the small number of patients we did not correct the *p*-values for multiple testing in the GA to smoking status analysis. Unsupervised hierarchical clustering based on smoking status was used to visualize GA frequencies amongst different smoking cohorts.
